# The future of surgical research

**DOI:** 10.1093/bjs/znaf095

**Published:** 2025-06-03

**Authors:** Michael Gnant

**Affiliations:** Comprehensive Cancer Center, Medical University of Vienna, Vienna, Austria; Austrian Breast & Colorectal Cancer Study Group, Vienna, Austria

There are global shortcomings in funding surgical research compared with other fields of medicine and these differences are particularly acute in clinical trials^1^. There is a clear imbalance between the underrepresentation of surgical clinical research compared to the ubiquity of surgical procedures globally and no impact has been made in the last two decades^2^.

Most clinical trials are funded by pharmaceutical or medical device companies with few being funded by governmental agencies. The consequences are a focus on commercially attractive innovations rather than large pragmatic clinical trials with endpoints that are either patient focused or have direct public health impact.

Publicly sponsored trials are more likely to focus on therapies for rare diseases, and survivorship and quality of life^3^. Despite the substantial cost of pivotal clinical (registration) trials, their relative share of the overall cost of drug development is modest^4^, and the perspective of a ‘return on investment’ is markedly more difficult outside the field of an innovative marketable drug or device. Clinical trials funded by governmental or philanthropic entities are perceived to be less biased, more transparent, and less commercially driven, but they do not appear to be any more impactful in clinical practice guidelines than company-sponsored trials^5^.

Surgery is often embedded in interdisciplinary trials such as in the field of cancer. Unfortunately, in these trials the potential to generate surgical knowledge is not fully realized. In part, this is because of missing harmonization of surgical documentation methods: any initiative to standardize and simplify the documentation of tools for surgical aspects of interdisciplinary clinical trials is therefore highly commendable^6^. In addition, several initiatives to specifically strengthen, fund, and support surgical clinical research have been conducted, with varying success^7^. Modern clinical trial ‘technology’, including innovative statistical methods and AI-driven optimization of trial design and outputs, will also be helpful in the future^8^.

In addition to assessing the pros and cons of commercial *versus* public funding of clinical trials^9^, it is also worthwhile taking a deeper dive into surgical culture and its traditions, in an attempt to identify potential inherent reasons for the described challenges for surgical research. Among all medical disciplines, surgery stands out in several ways. First, it is clearly a vital branch of medicine, often life-saving, sometimes spectacular, and it is an essential component of the treatment of cancer, cardiovascular disease, trauma, and infections. Second, due its nature of being invasive *en principe*, it necessitates (and creates) a special level of trust between patients and their surgeons. There clearly exists a difference between allowing someone to take a scalpel to cut them open compared to accepting the prescription of a new drug. The latter aspect may be one reason why surgeons sometimes consider themselves more as artists than technicians, researchers, or caregivers. Although this special relationship surgeons enjoy with their patients is one of the most rewarding characteristics of our profession, it also acts as a potential barrier to systematic and rational knowledge generation and can entice us to authoritarian behaviour and patronizing internal structures.

High-quality clinical knowledge generation relies on RCTs as a methodology for evaluating innovative therapeutic approaches, mainly based on the beauty and power of randomization to eliminate unknown confounders^10^. RCTs do, however, need careful planning, systematic thinking, collaboration with others; in short: a lot of effort including communication. The art of Medicine begins where standards end. The clinical application of knowledge in an individualized and empathic manner is built on the availability of evidence that can only be produced by appropriate research methodology, and there is no contradiction between any state-of-the-art and constant and recurring questioning of it. In fact, this is a matter of strategy for a whole discipline, how it creates and allows for scientific education and career planning, which for some surgeons includes time in the laboratory or other research facilities. Fewer surgeons in training are exposed to these opportunities, despite the clear observation that dedicated laboratory time as part of early career phases will highly determine whether a young surgeon will later become a funded researcher^11^. It is also an institutional obligation to create career paths that allow surgical fellows to engage in translational and clinical trials that might deliver results only many years later without immediate reward^12^. But above all, young surgeons must be taught to be alert, critical, resilient, reflecting, not always accepting the obvious, and questioning their mentors all the time.

Although clinical trials remain important, other sources of information have become more relevant in recent years. Abundant real-world data from patient registries and digital medical records are being exploited to drive evidence-based medicine. Real-world data provide excellent opportunities for surgical research, focusing on outcomes rather than solely on techniques and on the patient rather than necessarily the disease or surgical procedures^13^. With the inevitable advent of artificial intelligence in medicine, surgery will have to redefine itself. Synthesizing clinical experience and surgical knowledge to diagnose clinical conditions may soon be taken over by AI algorithms. However, identifying the right questions to ask, communicating the answers with patients and colleagues, accepting responsibility for the perioperative process, and striving for perfection will remain the domain of the surgeon.

Surgical standards are often based on strong opinions, entrenched hierarchies, and change has always been difficult. It is our responsibility to create surgical education systems that embrace research, critical thinking, and systematic evaluation of new ideas. Eventually, the fate of our whole surgical discipline will depend on whether we successfully tackle these challenges, regenerating research as a major principle of surgical development and careers and focusing on creating knowledge and eventually benefits for patients rather than on manual and technical aspects of our profession^14^.
